# Odontogenic carcinoma with dentinoid: case report and literature review of a rare entity

**DOI:** 10.1186/s12903-024-04471-8

**Published:** 2024-06-18

**Authors:** Ming Zeng, Xiaolong Guo, Xinming Chen, Zhe Shao, Shaodong Yang

**Affiliations:** 1 State Key Laboratory of Oral & Maxillofacial Reconstruction & Regeneration, Key Laboratory of Oral Biomedicine Ministry of Education, Hubei Key Laboratory of Stomatology, School and Hospital of Stomatology, Wuhan University, Wuhan, China; 2 Department of Oral Radiology, School & Hospital of Stomatology, Wuhan University, Wuhan, China; 3 Department of Pathology, School & Hospital of Stomatology, Wuhan University, Wuhan, China; 4 Department of Oral and Maxillofacial-Head and Neck Oncology, School & Hospital of Stomatology, Wuhan University, Wuhan, China

**Keywords:** Odontogenic carcinoma, Odontogenic tumor, Dentinoid, Mandible, Jaw

## Abstract

**Background:**

Odontogenic carcinoma with dentinoid (OCD) is a rare and controversial entity, which has not yet been included in the current World Health Organization classification of odontogenic lesions. Owing to the small number of reported cases, the clinicopathological characteristics, biological behavior, prognosis, and appropriate treatment strategies for OCD remain to be defined. Herein, we present an additional case of OCD with a focus on the differential diagnosis and review of the pertinent literature, in order to enable better recognition by oral clinicians and pathologists and further characterization of this entity.

**Case presentation:**

This paper reports a case of OCD in the posterior mandible of a 22-year-old female. Radiography showed a well-defined unilocular radiolucency with radiopaque materials. The intraoperative frozen section pathology gave a non-committed diagnosis of odontogenic neoplasm with uncertain malignant potential. Then a partial mandibulectomy with free iliac crest bone graft and titanium implants was performed. Microscopically, the tumor consisted of sheets, islands, and cords of round to polygonal epithelial cells associated with an abundant dentinoid matrix. Immunohistochemically, the tumor cells were diffusely positive for CK19, p63, and β-catenin (cytoplasmic and nuclear). No rearrangement of the *EWSR1* gene was detected. The final diagnosis was OCD. There has been no evidence of recurrence or metastasis for 58 months after surgery. We also provide a literature review of OCD cases, including one case previously reported as ghost cell odontogenic carcinoma from our hospital.

**Conclusions:**

OCD is a locally aggressive low grade malignancy without apparent metastatic potential. Wide surgical excision with clear margins and long-term period follow-up to identify any possible recurrence or metastases are recommended. Histopathological examination is essential to conclude the diagnosis. Special care must be taken to distinguish OCD from ghost cell odontogenic carcinoma and clear cell odontogenic carcinoma, as misdiagnosis might lead to unnecessary overtreatment. Study of additional cases is required to further characterize the clinicopathological features and clarify the nosologic status and biological behavior of this tumor.

**Supplementary Information:**

The online version contains supplementary material available at 10.1186/s12903-024-04471-8.

## Introduction

Odontogenic carcinoma with dentinoid (OCD) is a rare and controversial entity [1–4]. It was first introduced into the medical literature by Sawyer et al. [2] in 1986, and was later explained in more detail by Mosqueda-Taylor et al. [1] in 2014. Since then, only two more cases were reported with this nomenclature, highlighting its rarity [3, 4]. OCD has not yet been included in any current odontogenic tumor classifications. In the fourth edition of World Health Organization (WHO) classification in 2017, OCD was only briefly discussed under clear cell odontogenic carcinoma (CCOC), with the comment that dentinoid may be present in a small subset of CCOC generally as a minor inductive change, and those lesions with extensive dentinoid may represent a separate entity [5]. Although mentioned frequently in the differential diagnosis of other odontogenic tumors such as adenoid ameloblastoma (AdAM) and CCOC, OCD was still not recognized as an independent entity in the latest 2022 WHO classification [6–8].

OCD can occur in a wide age range (14–74 years), and usually presents as a painless swelling of the jaw with predilection for the posterior regions. Radiographs usually show well-defined radiolucencies, and variable amounts of radiopaque calcified material can be seen in some cases [1, 3]. Histopathologically, OCD is composed of sheets, nests and cords of eosinophilic, pale, or clear epithelial cells associated with prominent dentinoid material deposition in a slightly or mature myxoid connective stroma [1, 3]. It has a variable degree of cytological atypia, and may have focal areas with microcystic pattern, duct-like structures or ameloblast-like differentiation [1, 3, 4]. Occasionally, clusters of polyhedral epithelial cells with eosinophilic cytoplasm and squamoid features resembling calcifying epithelial odontogenic tumor (CEOT) may be focally present [1, 3]. OCD typically shows evidence of tumor infiltration into adjacent tissues, including the presence of perineural invasion in some tumors, however, reports on metastasis are lacking [1–4]. While only two OCD cases have been tested, recent genetic data has shown the pathogenic mutations in CTNNB1 and APC genes, which are both part of the Wnt/β-catenin signaling pathway, present in this tumor [3]. Consistent with Wnt/β-catenin pathway activation, these two tumors showed strong β-catenin accumulation in the cytoplasm and in the nuclei [3].

Owing to the small number of reported cases so far, the clinicopathological characteristics, biological behavior, prognosis, and appropriate treatment strategies for OCD remain to be defined. Herein, we present an additional case of OCD with a focus on the differential diagnosis and review of the pertinent literature, in order to enable better recognition by oral clinicians and pathologists and further characterization of this entity.

## Case report

**Fig. 1:**
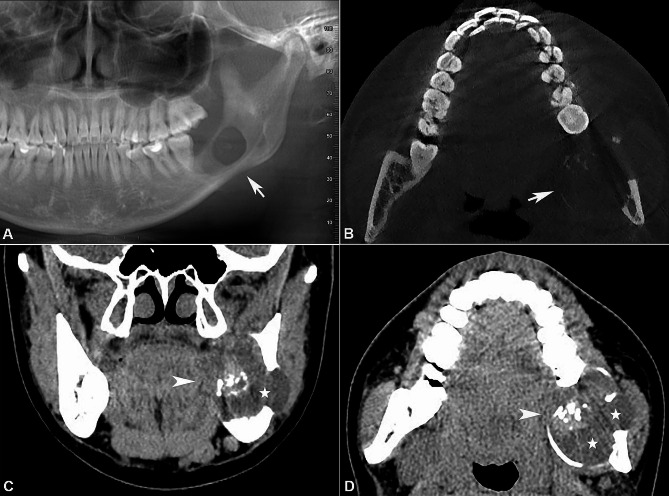
Radiographic findings. Reconstructed panoramic view (**A**) and axial view (**B**) of CBCT images showed a unilocular, well-defined radiolucency with radiopaque materials in the left posterior mandible, with significant expansion and perforation of the buccal and lingual cortical plates (arrow). Coronal view (**C**) and axial view (**D**) of MDCT images with soft tissue algorithm demonstrated a well-delineated, heterogeneous mass consisting of solid (arrow head) and cystic (star) areas. Radiopaque calcifications were prominent

In March 2019, a 22-year-old female patient presented with a painless swelling in the left posterior mandible that had been present for approximately six months. She had undergone tooth extraction in the same region by a community dentist nine months earlier, but without prior radiographic examination. Intraroral examination showed a firm swelling in the left retromolar pad area covered by a normal to erythematous mucosa, with an ulcerated area due to occlusal trauma. Extraoral examination revealed no evident facial asymmetry. There were no palpable cervical lymph nodes and no paresthesia. The medical and family histories were unremarkable. Cone beam computed tomography (CBCT) scan revealed a unilocular well-defined radiolucency with radiopaque foci, extending from the left mandibular second molar to the ascending ramus of the left mandible (Fig. 1A and B). Significant expansion of the left mandible and perforation of the buccal and lingual cortical plates were noted. Multidetector row computed tomography (MDCT) (BrightSpeed; GE Healthcare, Waukesha, WI) scan showed a well-delineated, heterogeneous mass measuring 4.4 × 4.3 × 4.0 cm, consisting of solid and cystic areas; radiopaque calcifications were noted within the solid areas (Fig. 1C and D). The clinical and radiographic appearances suggested a benign odontogenic tumor. Intraoperative frozen section was used; a non-committed diagnosis of odontogenic neoplasm with uncertain malignant potential was rendered. Subsequently, a partial mandibulectomy with free iliac crest bone graft and titanium implants was performed.

**Fig. 2:**
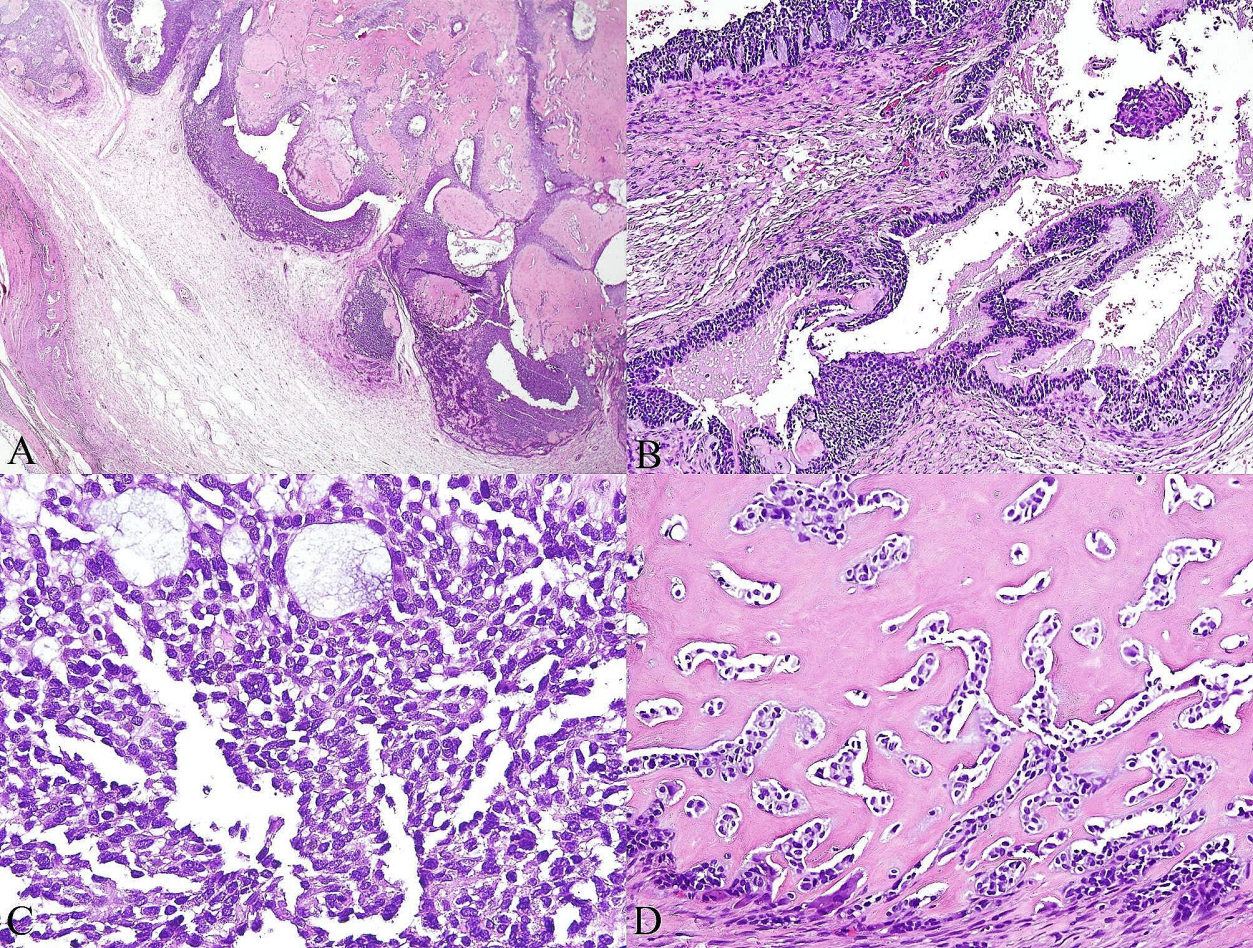
Histopathological findings. **A**, Low power view showing the tumor consisting of sheets and islands of epithelial tumor cells associated with an abundant dentinoid matrix (hematoxylin-eosin [H&E], original magnification ×20). **B**, Cystic degeneration of epithelial islands (H&E, original magnification ×100). **C**, Hyperchromatic basaloid cells with scant to moderate amounts of pale eosinophilic cytoplasm and a high nuclear: cytoplasmic ratio (H&E, original magnification ×400). **D**, Some tumor cells approximating the dentinoid showing plasmacytoid morphology, with abundant eosinophilic cytoplasm and eccentric nuclei (H&E, original magnification ×200)

**Fig. 3:**
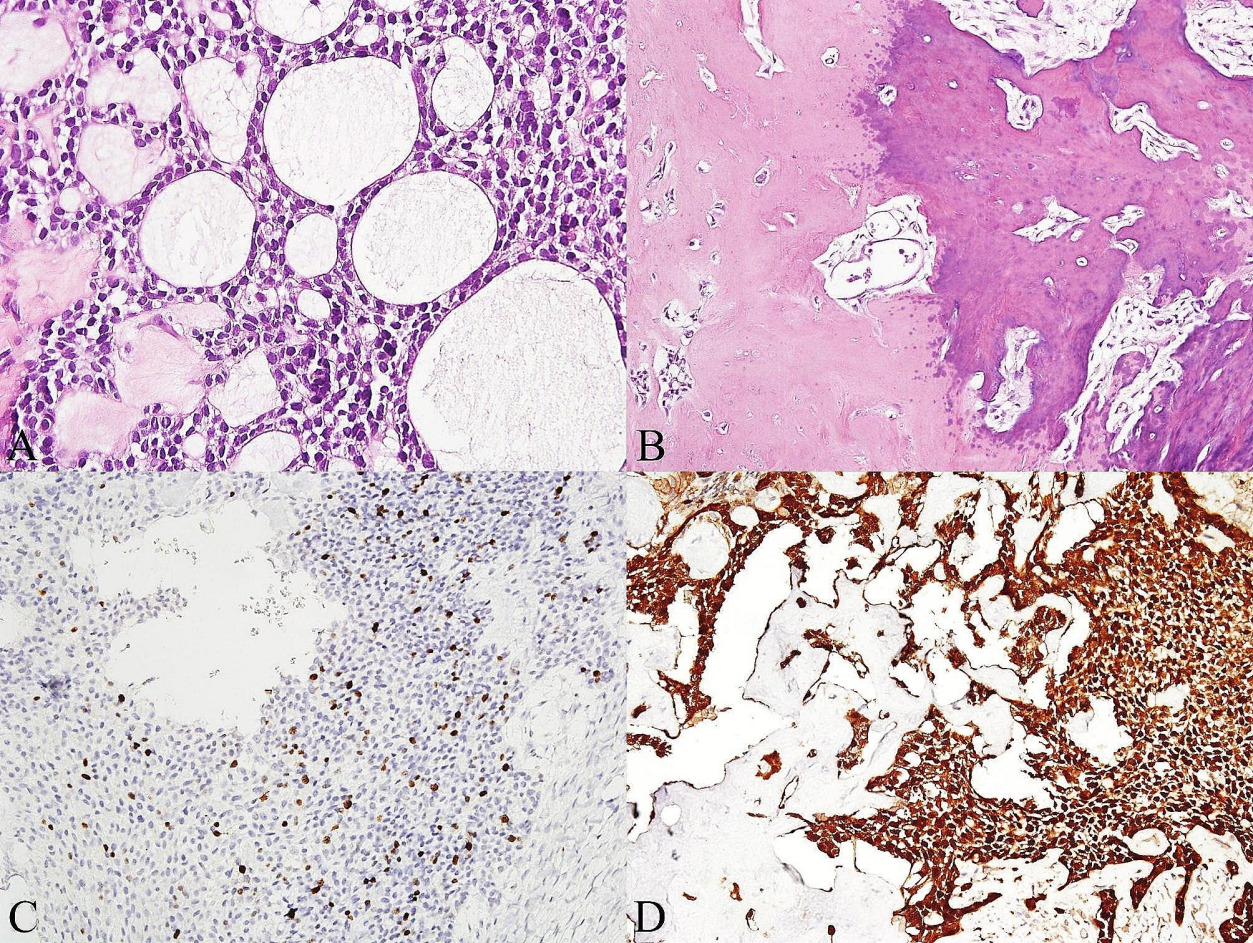
Histopathological and immunohistochemical findings. **A**, In some areas the tumor cells displaying prominent clear cell cytoplasm and microcystic change (H&E, original magnification ×400). **B**, Eosinophilic dentinoid with globular calcification (H&E, original magnification ×200). **C**, The Ki67 proliferation index was about 10% (original magnification ×200). **D**, Tumor cells showing strong and diffuse cytoplasmic and nuclear positivity for β-catenin (original magnification ×200)

Gross examination of the specimen revealed a firm mass of the left posterior mandible; the cut surface was mainly solid with partially cystic spaces and yellowish-white in color. Microscopically, the tumor consisted of sheets, islands, and cords of hyperchromatic basaloid cells associated with an abundant dentinoid matrix (Fig. 2A). Cystic degeneration of epithelial islands was frequently observed, and in some areas necrosis was observed in the superficial aspect of the epithelial lining (Fig. 2B). On high power magnification, most tumor cells contained scant to moderate amounts of pale eosinophilic cytoplasm and indistinct cell borders, with round to oval hyperchromatic nuclei, dispersed chromatin and inconspicuous nucleoli (Fig. 2C). Moderate nuclear atypia was present, but mitotic figures were scarce. Tumor cells that were adjacent to or intimately intermingled with the dentinoid material had more eosinophilic cytoplasm, and some were round to plasmacytoid in appearance (Fig. 2D). In some areas, the tumor cells displayed prominent clear cell cytoplasm (Fig. 3A). Microcyst formation was occasionally seen within the epithelial islands, focally creating a cribriform appearance (Fig. 3A). Vague peripheral palisading in the epithelial islands were noted in some areas. Neither true ducts nor ghost cells were observed. Perineural invasion and lymphovascular invasion were not noted. The eosinophilic masses of dentinoid had occasional cell inclusions, and irregular tubules were identified in some areas. Some of these dentinoid masses showed evidence of globular foci of mineralization (Fig. 3B). Immunohistochemically, the tumor cells were diffusely positive for CK19 and p63, and negative for CD56, calretinin, and p53. The Ki67 proliferation index was estimated to be about 10% (Fig. 3C). The immunohistochemical study for β-catenin showed strong and diffuse cytoplasmic and nuclear positivity (Fig. 3D). No rearrangement of the *EWSR1* gene was detected by fluorescence in situ hybridization using the *EWSR1* (22q12) dual-color break-apart probe (Anbiping, Guangzhou, China). A final diagnosis of OCD of the mandible was established.

The surgical resection margins were free of tumor, and no additional treatment was performed postoperatively. The patient was followed up for 58 months, with no sign of recurrence or metastasis.

## Literature review

An electronic search in PubMed, without time or language restrictions, was performed up to May 1st, 2024. The keywords “odontogenic carcinoma with dentinoid”, “odontogenic carcinoma with osteodentin”, and “odontogenic carcinoma with dentin”, were used. Given only 27 results obtained in this initial search, a literature search was further conducted using the keywords “odontogenic tumor with dentinoid”, “odontogenic tumor with osteodentin”, and “odontogenic tumor with dentin” in order to identify relevant cases of OCD, which yielded 377 results. OCD cases included in this review met the following histopathological criteria:

1. An odontogenic tumor predominantly composed of eosinophilic, pale, or clear epithelial cells within a background of mature fibrous connective tissue.
2. Prominent dentinoid (dysplastic dentin or osteodentin) formation in close contact with neoplastic epithelium.
3. Presence of mild to severe cellular atypia and variable mitotic activity.
4. Presence of tumor infiltration into adjacent tissues; tumor necrosis, neural invasion, lymphovascular invasion, or metastasis, if present, as desirable features for the diagnosis of OCD.
5. The tumor does not fit into any defined tumor entity based on the current 5th edition of the WHO classification of odontogenic lesions [6].

Cases with insufficient/unconvincing histomorphological description or photographic evidence were excluded. The cases excluded from the present review but included in prior reviews [1, 3, 4] were discussed further below.

In their study, Mosqueda-Taylor et al. [1] presented a series of three cases of OCD and identified six additional similar cases from the literature before 2014. However, one should note here that reliable identification from the previously published cases based on the limited descriptions and figures is sometimes difficult or even impossible. A good example of this dilemma is the case of Ide et al. [9], which was included in the review by Mosqueda-Taylor et al. [1] as an example of OCD. This tumor was reclassified as sclerosing odontogenic carcinoma later by Ide et al. [10] and other authors [11]. We agree that this lesion is not an OCD only because of the focal presence of dentinoid deposition as shown in the published photomicrographs [9, 10]. Among the other five cases regarded as earlier examples of OCD by Mosqueda-Taylor et al. [1], three cases were reported under the name of CCOC or clear cell odontogenic tumor (previously used terms for CCOC) [12–14]. However, information about the degree (focal or diffuse) of dentinoid formation in these cases was not available. In addition, these cases were included in the review of CCOC by Loyola et al. [15], reflecting different opinions about the diagnosis. Moreover, the case reported by Ariyoshi et al. [14] in 2002 exhibited clusters of ghost cells interspersed among the clear cell component; the authors discarded the possibility of a ghost cell odontogenic carcinoma (GCOC) because no epithelial lining compatible with calcifying odontogenic cyst was found and because there are no reported cases of odontogenic ghost cell lesions with a clear cell component at that time. However, soon afterwards a case of dentinogenic ghost cell tumor (DGCT) with prominent clear cell components was reported by Yoon et al. [16], who considered that the tumor reported by Ariyoshi et al. [14] might represent a clear variant of ghost cell odontogenic tumor. Therefore, despite being included in the prior review by Mosqueda-Taylor et al. [1], these four cases, were excluded in the present review because of the diagnostic uncertainty.

One case reported as a clear cell cystic variant of CEOT containing abundant dentinoid material by Urias Barreras et al. [17] in 2014 was not included in prior reviews for OCD [3, 4]. Based on the morphological description and the images provided in the manuscript, there were no areas with typical CEOT morphology. As mentioned below, the multifocal amounts of amyloid seen in this tumor do not necessarily support the diagnosis of CEOT. Moreover, although focal dentinoid deposition can be seen in a wide variety of odontogenic lesions, the presence of large amounts of dentinoid material is not a feature of CEOT. Therefore, it is our opinion that this tumor might actually represent OCD. We therefore included this case in the present review.

Overall, our literature survey disclosed nine cases that met our study criteria for OCD (Table 1) [1–4, 17–19].

**Table 1:** Clinical and radiographic features of odontogenic carcinoma with dentinoid

Case	Author/year	Sex /age (y)	Site	Size (cm) #	Radiographic findings	Treatment	Original diagnosis	Recurrence	Follow-up and outcome
1	Sawyer et al. [2], 1986	M/14	Maxilla (molar region)	NA	Well-defined radiolucency with patchy areas of radiopacity	Enucleation (1st) Marginal resection (2ed)	Odontogenic carcinoma with dentinoid	One (at 1 year)	18 months NED
2	Punnya et al. [18], 2004	F/18	Maxilla (premolar region)	NA	Well-defined radiolucency with small radiopaque foci and root resorption	Enucleation	Primary intraosseous odontogenic carcinoma	No	16 months NED
3	Urias Barreras et al. [17], 2014	M/31	Mandible (molar region and ramus)	3.0 × 3.0 × 2.0	Well-defined, unilocular radiolucency with central radiopacity and root resorption	Tumor excision	Calcifying epithelial odontogenic tumor, clear cell cystic variant	NA	NA
4	Mosqueda-Taylor et al. [1], 2014	M/34	Mandible (third molar and ramus)	2.0 × 2.0	Well-defined radiolucency with central radiopacity	Tumor excision with extraction of third molar (1st); Mandibular resection with left neck lymph node dissection (2ed)	Odontogenic carcinoma with dentinoid	One (at 42 months)	Lost to follow-up
5	Mosqueda-Taylor et al. [1], 2014	M/31	Mandible (molar region and ramus)	NA	Well-defined, multilocular radiolucency with large radiopaque foci	Segmental resection	Odontogenic carcinoma with dentinoid	No	2 years NED
6	Mosqueda-Taylor et al. [1], 2014	F/32	Maxilla (premolar region)	NA	Well-defined, unilocular radiolucency with small radiopaque foci	Partial maxillectomy	Odontogenic carcinoma with dentinoid	No	3 years NED
7	Gondak et al. [3] 2020 and Mariano et al. [4], 2020	M/41	Maxilla	NA	Extensive, well-defined, radiolucency (from the second recurrent tumor)	Curettage (1st); Surgical resection (2ed); Surgical resection followed by radiotherapy and chemotherapy (3rd)	Odontogenic carcinoma with dentinoid	Yes, 2 times (at 7 and 9 years after initial treatment)	2 years NED
8	Gondak et al. [3], 2020	F/74	Maxilla (unerupted canine to posterior region)	4.0 × 3.0	Multilocular radiolucency containing small radiopaque foci	Enucleation (1st); Wide resection (2ed)	Odontogenic carcinoma with dentinoid	One (at 2 years)	3 years NED
9	Ullah et al. [19], 2022	M/39	Maxilla (posterior region)	3.75 × 2.4 × 2.84	A heterogeneous soft tissue mass (from the recurrent tumor)	Tumor excision (1st); Right maxillectomy and right neck dissection. four months of adjuvant radiation (2ed)	Clear cell odontogenic carcinoma	One (at 1 year)	One year NED
10	Cheng et al. [20], 2004	M/44	Mandible (molar region )	NA	Well-defined, unilocular, radiolucency with root resorption	Enucleation (1st); Enucleation (2ed); Surgical resection (3rd); Surgical resection (fourth); Partial mandibulectomy (fifth)	Ghost cell odontogenic carcinoma	Yes, 4 times (at 1, 4, 8 and 13 years after initial treatment).	Lost to follow-up
11	Present case	F/22	Mandible	4.4 × 4.3 × 4.0	Well-defined, unilocular, radiolucency with small radiopaque foci	Partial mandibulectomy	Odontogenic carcinoma with dentinoid	No	Four years and ten months NED

M, male; F, female; NA, not available; NED, no evidence of disease; # Size of the lesion estimated through radiographic images

## Discussion

OCD is a rare and poorly characterized odontogenic tumor, which has not been formally included in the two most recent editions of the WHO classification of Head and Neck Tumors [5, 6]. Due to its recent description and rarity, few pathologists and perhaps fewer clinicians are familiar with this entity, and it is likely underrecognized and under-reported. As noted by Mosqueda-Taylor et al. [1], OCD may have been classified or misdiagnosed as other odontogenic neoplasms, such as CCOC and primary intraosseous odontogenic carcinoma. This study reports on an unusual odontogenic tumor microscopically characterized by abundant dentinoid formation in association with the neoplastic odontogenic epithelium. The features of this lesion do not fit well in any odontogenic tumor category according to the latest 2022 WHO classification, but are highly similar to those of OCD cases previously described by Mosqueda-Taylor et al. [1]. In addition, in our recent retrospective review of odontogenic ghost cell lesions, we identified another case of OCD from our hospital, which was previously reported as GCOC (case 4 in the study by Cheng et al. [20]). This patient was a 44-year-old male, who presented with a painless swelling in the right posterior mandible for 18 months. The patient was initially treated with enucleation. The original pathological diagnosis was CEOT, probably due to the pathologist’s misinterpretation of the dentinoid as amyloid. The lesion recurred four times over a span of 13 years. The pathologist reported the lesions in the first and second recurrences to be consistent with a recurrent CEOT. In the third recurrence, the diagnosis was revised and modified to atypical ameloblastoma. In the fourth recurrence, 13 years after the initial diagnosis, the tumor was revised again and it was then classified as GCOC, mainly due to the presence of some ghost cells and perineural invasion (Fig. S1). However, the isolated small foci of ghost cells were only noted in one histological section of the fourth recurrent tumor, which was not a significant finding to consider a diagnosis of odontogenic ghost cell lesions. At this point, the patient was lost to follow-up and the ultimate fate is not known. Of note, this patient had the longest follow-up time (13 years) in the literature for OCD, but neither metastasis nor histologically high-grade transformation was noted.

Therefore, our literature review along with the two cases from our hospital yielded a total of 11 cases of OCD (Table 1) [1–4, 17–20]. The mean age of the patients was 34.5 years, with a range between 14 and 74 years. Seven cases affected males and four occurred in females, with a male to female ratio of 1.75:1. Six tumors occurred in the maxilla and five in the mandible. A remarkable predilection for the posterior regions (100%) of the jaws was observed. Of the nine primary lesions with imaging examination results, all appeared as well-defined radiolucencies, and eight contained variable amounts of mineralized material; seven lesions were described as unilocular and two as multilocular. All four recurrent lesions with available radiographic examination appeared as radiolucent lesions, three of which were with ill-defined margins [1, 3, 4, 19, 20]. Surgery was the primary therapeutic approach in all cases, including conservative (simple excisions, curettages, and enucleations) or radical (partial and segmental resections) modalities. Two patients underwent neck dissection at the time of recurrent tumor resection, which did not show any evidence of regional lymphatic metastasis [1, 19]. Postoperative radiotherapy and chemotherapy was performed in one patient [3, 4], and adjuvant radiotherapy was performed in another patient [19]; in the latter case, the authors considered OCD to be synonymous with CCOC with dentinoid [19]. Follow-up information was available in 10 of 11 patients, however, the follow-up time of nine patients was shorter than 5 years. Recurrence was recorded in six patients, all of which were initially treated with conservative surgical methods [1–4, 19, 20]. No regional or distant metastases were found in any patient [1–4, 17–20].

As the clinical and radiographic features of OCD are non-specifc, histopathological examination is essential to conclude the diagnosis. As the name implies, a signature feature of OCD is the prominent dentinoid formation associated with epithelial tumor cells, but this feature is not pathognomonic. Dentinoid may be seen in many other already defined odontogenic tumors, which should be excluded before rendering an OCD diagnosis [1, 15, 21–26]. Among them, DGCT and AdAM frequently contain significant amounts of dentinoid deposition in close contact with neoplastic odontogenic epithelium and GCOC can contain variable dentinoid material, thus being the main histological differential diagnoses of OCD [21, 23–26].

DGCT is a rare benign but locally invasive odontogenic tumor, generally considered to be the solid, neoplastic counterpart of calcifying odontogenic cyst, while GCOC was classified as the malignant counterpart of calcifying odontogenic cyst and DGCT [21, 23–26]. AdAM is a novel epithelial odontogenic neoplasm recently included in the new fifth edition of the WHO classification of odontogenic tumors [6–8]. Essential diagnostic features for AdAM include epithelium resembling conventional ameloblastoma, duct-like structures, whorls/morules, and cribriform architecture. Dentinoid, clear cells, and focal ghost cell keratinization are desirable diagnostic criteria for AdAM [6–8, 21, 24–26]. Recent genetic studies have shown that both AdAMs and DGCTs are characterized by frequent activation of the Wnt/β-catenin signaling pathway, which has also been reported to occur in OCDs [3, 21, 25, 26]. Thus, differential diagnosis between these tumors from a genetic standpoint is difficult. In line with this, nuclear expression of β-catenin is a common feature of these three types of lesions and has no utility in the distinction. The shared alterations of the Wnt/β-catenin signaling pathway also suggested a possible relationship among OCD, DGCT and AdAM, which may belong to the same tumor spectrum [21, 24–26]. Recently Oh et al. proposed the unifying terminology of ‘Wnt pathway-altered benign odontogenic tumor’ to encompass DGCT and AdAM [21]. In their study, they also included a single case of odontogenic tumor characterized by the diffuse presence of dentinoid but without adenoid structures or ghost cells. The overall features of this lesion were similar to those of OCD, but it was classified as a benign odontogenic tumor by Oh et al. [21].

By definition, the presence of abundant ghost cells is a definite requirement for the diagnosis of both DGCT and GCOC [6–8, 23]. In the present case, the absence of ghost cells readily ruled out DGCT and GCOC. Difficulties may arise when occasional ghost cell keratinization is present in an OCD. In addition to the family of odontogenic ghost cell lesions (calcifying odontogenic cyst, DGCT and GCOC), ghost cells have been described in a variety of other odontogenic lesions [21, 24–26]. However, while ghost cells are prevalent in odontogenic ghost cell lesions, it is only focally on OCD when they appear [1], as exemplified by the above-mentioned case from our hospital (Fig. S1). To date, no reliable diagnostic criteria in distinguishing AdAM from OCD have been established. Pseudocyst structures and columnar cells with prominent palisading can be focally present in OCDs, as seen in the present case, making the distinction challenging [1, 3, 4]. However, considering the minimal epithelium resembling conventional ameloblastoma, the presence of moderate cytological atypia, and the absence of epithelial whorls/morules, the present case does not meet the strict diagnostic criteria of AdAM.

Another important differential diagnosis of OCD is CCOC, which is a rare odontogenic carcinoma histologically characterized by nests, sheets, and cords of glycogen-rich clear cells and cells with eosinophilic cytoplasm in a fibrocellular or hyalinized stroma [15, 27, 28]. The ratio of clear to eosinophilic cells in CCOC varies from case to case, and the predominance of eosinophilic cells may be seen in a minority of cases. Similarly, the tumor cell population of OCD is often biphasic, consisting of cells with clear or eosinophilic cytoplasm. Columnar cells showing evidence of ameloblastic differentiation on the periphery of tumor cell islands may be present in both entities. The description of CCOC cases with dentinoid deposition in the literature further complicates the distinction between OCD and CCOC [12–15, 19]. According to a systematic review by Loyola et al. [15] in 2015, dentinoid depositions were seen in 7 of 94 cases (7.4%) of CCOC. However, three of the seven tumors were regarded as earlier examples of OCD by Mosqueda-Taylor et al. [1]. While the presence of extensive dentinoid should trigger consideration of OCD, it is believed that focal dentinoid deposition can be present in CCOC, as seen in so many other odontogenic tumors [1, 5, 22]. However, the degree of dentinoid formation that may distinguish between OCD and CCOC is unclear at present. Fortunately, molecular testing can aid in distinguishing CCOC from OCD. Genetically, CCOCs harbor EWSR1 rearrangements with *CREB* family members, most commonly *EWSR1::ATF1* and rarely *EWSR1::CREB1* or *EWSR1::CREM* gene fusions [27, 28]. So far, no mutations in the genes of Wnt/β-catenin signaling pathway have been recorded in CCOCs. In the present case, rearrangement for *EWSR1* was tested for exclusion of CCOC and no rearrangement was found. On the other hand, extensive dentinoid and nuclear expression of β-catenin were present in our case, favoring the diagnosis of OCD.

In most instances, distinction between CEOT and OCD is straightforward based on morphology. Given the predominance of clear cells in some OCD cases, the clear cell subtype of CEOT should especially be considered in the differential diagnosis [17]. The presence of foci of classic CEOT is a useful clue to the diagnosis of clear cell subtype of CEOT. On the other hand, the presence of eosinophilic dentinoid is the striking feature of OCD, but uncommonly reported in CEOT. In difficult cases or small biopsies, the presence of nuclear β-catenin by immunohistochemistry could be helpful in distinguishing OCD from CEOT. Two cases of CEOT examined for β-catenin lacked nuclear immunoreactivity [29, 30]. The presence of amyloid is considered characteristic of CEOT, but it should also be noted that such material is not specific for CEOT [31, 32]. A recent study by Al-Qazzaz et al. [32] revealed that, in addition to CEOT, various other odontogenic cysts and tumors, such as calcifying odontogenic cyst, ameloblastoma, adenomatoid odontogenic tumor, DGCT, and amyloid-rich subtype of odontogenic fibroma also produce amyloid in varying proportions. In this study, multifocal amounts of amyloid were detected in one of the two DGCT cases tested [32]. Considering the potential relationship between DGCT and OCD, it seems plausible that focal or multifocal amounts of amyloid could be present in some OCDs. As mentioned above, we believe that the morphological phenotype of the tumor reported by Urias Barreras et al. [17] more closely matches OCD rather than CEOT, and the presence of multifocal amounts of amyloid in this tumor should not be considered a diagnosis of exclusion of OCD.

Given the small number of reported cases of OCD and limited clinical follow-up, it is not possible to reliably comment on its behavior and prognosis. At present, it would seem prudent to manage OCD in a similar manner to other locally aggressive odontogenic tumors or low-grade odontogenic carcinomas, i.e., complete resection with negative margins. Neck dissection might be unnecessary, as no report to date has disclosed lymph node metastases [1–4, 17–20]. There is no evidence that adjuvant therapies, such as chemotherapy or radiotherapy, are necessary in addition to surgery. From this point of view, distinguishing OCD from DGCT and AdAM does not seem to be clinically important. However, distinguishing OCD from CCOC and GCOC is of paramount importance, as the latter two entities present with metastasis in 31% and 16.7% of cases, respectively, and both have shown the capacity for distant metastases [15, 23].

## Conclusions

In conclusion, OCD is a rare and poorly characterized odontogenic tumor, which may have been classified or misdiagnosed as other odontogenic neoplasms because of a lack of awareness and its relatively recent description. Histopathological examination is essential to conclude the diagnosis. Special care must be taken to distinguish OCD from CCOC and GCOC, as misdiagnosis might lead to unnecessary overtreatment, a situation that has been reported previously. Based on the limited data, OCD has not shown a tendency to metastasize; thus, wide surgical excision with clear margins and long-term period follow-up to identify any possible recurrence or metastases are recommended. Study of additional cases is required to further characterize the clinicopathological features and clarify the nosologic status and biological behavior of this tumor.

### Electronic supplementary material

Below is the link to the electronic supplementary material.

Supplementary Material 1

## Data Availability

No datasets were generated or analysed during the current study.

## Abbreviations

OCD: Odontogenic carcinoma with dentinoid
CCOC: Clear cell odontogenic carcinoma
AdAM: Adenoid ameloblastoma
CEOT: Calcifying epithelial odontogenic tumor
GCOC: Ghost cell odontogenic carcinoma
DGCT: Dentinogenic ghost cell tumor
WHO: World Health Organization
