# Supplementary material for: Odontogenic carcinoma with dentinoid: case report and literature review of a rare entity
Source: BMC Oral Health. 2024 Jun 18;24:704. doi: 10.1186/s12903-024-04471-8 (PMC11184837; doi:10.1186/s12903-024-04471-8)
Supplement: Supplementary file 1 — Supplementary Material 1 [file 12903_2024_4471_MOESM1_ESM.docx]

**Additional file 1 of Odontogenic carcinoma with dentinoid: case report and literature review of a rare entity**

**
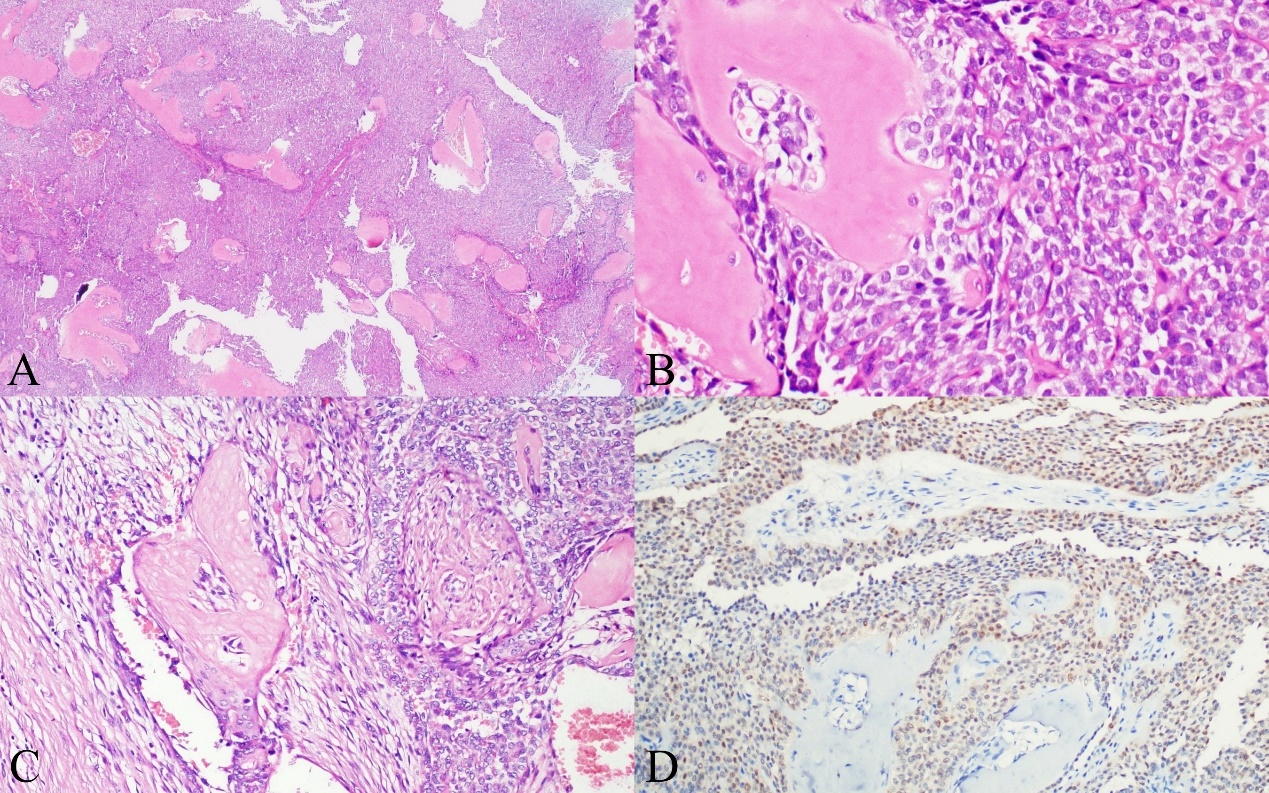
**

**Fig. S1.** Histopathological and immunohistochemical findings of the case previously reported as ghost cell odontogenic carcinoma (case 4 in the study by Cheng et al. [9]). A, Low power view showing the primary tumor consisting of sheets of epithelial tumor cells with copious amounts of dentinoid (H&E, original magnification ×40). B, Tumor cells with clear to eosinophilic cytoplasm and vesicular or hyperchromatic nuclei (H&E, original magnification ×400). C, Clusters of ghost cells and perineural invasion were identified in the fourth recurrent tumor (H&E, original magnification ×200). D, Immunohistochemical investigation of the fourth recurrent tumor for β-catenin revealing cytoplasmic and nuclear positivity in most tumor cells (original magnification ×200).
